# *TTN*:c.12478del in proximal I-band of titin represents a common molecular cause of dilated cardiomyopathy in Slovenian patients

**DOI:** 10.1186/s13023-025-03613-7

**Published:** 2025-02-28

**Authors:** Nina Vodnjov, Andraž Cerar, Aleš Maver, Borut Peterlin, Karin Writzl

**Affiliations:** 1 Clinical Institute of Genomic Medicine, University Medical Centre Ljubljana, Ljubljana, Slovenia; 2 Biotechnical Faculty, University of Ljubljana, Ljubljana, Slovenia; 3 Department of Cardiology, University Medical Centre Ljubljana, Ljubljana, Slovenia; 4 Faculty of Medicine, University of Ljubljana, Ljubljana, Slovenia; 5 European Reference Network for Rare, Low Prevalence, Or Complex Diseases of the Heart (ERN GUARD-Heart), Amsterdam, The Netherlands

**Keywords:** Titin, *TTN*tv, I-band, *TTN*:c.12478del, Dilated cardiomyopathy, DCM

## Abstract

**Background:**

Titin truncating variants (*TTN*tv-s) are the most common genetic cause of dilated cardiomyopathy (DCM). Only rare *TTN*tv-s in the constitutively expressed exons of the A-band of the protein titin are associated with DCM according to the guidelines, however, studies in large cohorts of patients with DCM suggest that the region where *TTN*tv-s are associated with DCM is wider, extending at least into the I-band. The aim of this study was to describe the molecular pathology of *TTN*tv-s in Slovenian patients with cardiomyopathy and to clinically characterise the most recurrent *TTN*tv.

**Results:**

We collected all *TTN*tv-s identified in patients with cardiomyopathy using next-generation sequencing genetic testing between 2010 and July 2024, resulting in 42 unique variants identified in 54 patients. The *TTN*:c.12478del variant, affecting not the A-band but the proximal I-band, specifically the cardiac-specific N2Bus region, was found to be the most recurrent variant, present in seven (11.6%) probands with DCM. Genetic characterisation revealed a probable founder origin of the variant. Clinical characterisation of these probands revealed a phenotype consistent with DCM and severely reduced left ventricular ejection fraction in all probands. Three (43%) of the probands had atrial fibrillation and/or non-sustained ventricular tachycardia. Based on literature reports and evidence supporting the pathogenicity of the *TTN*:c.12478del variant affecting the proximal I-band, we classified all rare *TTN*tv-s in constitutively expressed exons of the I-band as (likely) pathogenic. Therefore, 33 (78.6%) *TTN*tv-s were classified as (likely) pathogenic (13 in the I-band, affecting 19 probands and 20 in the A-band affecting 25 probands), meaning that *TTN*tv-s were identified in 44 genotype-positive Slovenian probands with DCM, explaining 73.3% of the molecular pathology of DCM.

**Conclusion:**

We report an almost threefold higher diagnostic yield of *TTN*tv-s in probands with DCM compared to previously reported findings in cohorts of patients with DCM from other populations. We also highlight the need for screening for rare *TTN*tv-s in the constitutively expressed exons of the I-band and for *TTN*:c.12478del in patients with DCM in this geographical region.

## Background

Dilated cardiomyopathy (DCM) is associated with a genetic cause in 20–50% of patients [1], with the most common pathogenic variants associated with DCM identified in approximately 17% of genotype-positive probands being heterozygous titin truncating variants (*TTN*tv-s) [2]. *TTN*tv-s are also identified in ∼ 2% of gnomAD individuals from the control/biobank population (version 3.1.2; 306 individuals with *TTN*tv-s out of 16,465 individuals) [3, 4]. Diagnostic challenge of distinguishing between benign and pathogenic *TTN*tv-s remains due to the known incomplete penetrance of the pathogenic *TTN*tv-s.

Titin is a large protein that extends from the Z-disc through the I- and A-bands to the M-band of the sarcomere and plays an important role in cell structure and elasticity [5, 6]. Titin undergoes alternative splicing, with most variations occurring in the I-band region, where the N2BA and N2B isoforms have been found to predominate in the heart, whereas the N2A isoform prevails in skeletal muscle [7]. Both cardiac isoforms contain a specific N2Bus region, located in exon 48 according to the meta-transcript count [8], which has been shown to be important in providing flexibility as well as binding sites for signalling proteins [9–11].

Since rare *TTN*tv-s in constitutively expressed exons have been found to be strongly linked with DCM, studies in both control populations and patients with DCM indicate that the location and frequency of the *TTN*tv may be a discriminator [8, 12–17]. It is widely accepted that rare *TTN*tv-s in the constitutively expressed exons of the A-band [15–18] of the protein titin are causal for DCM. However, there is conflicting evidence on whether this finding applies exclusively to the A-band or extends to the broader gene region [8, 12–14].

The present study aimed to search the internal database to extract rare *TTN*tv-s in constitutively expressed exons identified in probands with cardiomyopathy. Our objectives were to describe the population-specific molecular pathology of *TTN* variants and to phenotypically characterise the recurrent *TTN*:c.12478del that is located in the proximal region of the I-band.

## Materials and methods

An in-house registry of Mendelian disorders was used to identify probands with cardiomyopathy who were reported to have rare *TTN*tv-s in constitutively expressed exons of the gene. The *TTN*:c.12478del variant was the most frequently identified in the probands and segregated in their relatives, and its origin was investigated by haplotype analysis. Probands with the variant and their relatives provided retrospective clinical and radiological data.

### Study cohort

Exome/genome sequencing data from approximately 15,000 probands are part of the Mendelian disease registry of genetic test results maintained by the Clinical Institute of Genomic Medicine (CIGM), Ljubljana. We searched for all probands with cardiomyopathy who were referred between January 2010 and July 2024. Sequencing and further bioinformatic data analysis were performed as previously described [19, 20]. Most of the probands were Slovenian, with a smaller proportion coming from neighbouring Balkan countries. We identified 569 probands, referring 268 (47.1%) for hypertrophic cardiomyopathy (HCM), 211 (37.1%) for DCM, 53 (9.3%) for arrhythmogenic cardiomyopathy (ACM), 31 (5.4%) for non-compaction cardiomyopathy (NCC), and 6 (1.1%) for restrictive cardiomyopathy (RCM).As this was a retrospective study, we have used the classification of cardiomyopathies that has been used at the time of referral. Overall, likely pathogenic or pathogenic variants (LP/P) in cardiomyopathy-related genes were identified in 81 (30.1%) probands with HCM, 60 (27.1%) with DCM, 16 (30.2%) with ACM, and 8 (25.8%) with NCC. The test results were screened for all reported variants in *TTN* (NM_001267550.2) that were either rare or absent in control populations, were located in constitutively expressed exons of the gene, and predicted to cause the truncation of the titin protein (frameshift, nonsense, and disrupting exon splice site variants) (*TTN*tv-s). Variants were defined as rare if their minor allele frequency was less than 0.01 in the gnomAD control populations [3] and located in constitutively expressed exons if the percentage splice index (PSI) of the exon was 90% or greater [8]. We identified 54 probands with *TTN*tv-s and included them in the study.

Additionally, a relative in whom *TTN*:c.12478del was identified by Sanger sequencing to investigate the haplotype surrounding the *TTN*:c.12478del was also included in the study.

### Sequencing and bioinformatics analysis

All probands underwent genetic testing in the form of exome sequencing and data analysis as previously described [19, 20]. The median minimum exome coverage was 100x, and over 98% of targets had at least 10x coverage. The collected data were re-interpreted prior to inclusion in the study, and the PanelApp Dilated Cardiomyopathy and Conduction Defects v.82 virtual panel was used to screen for pathogenic variants in genes associated with DCM. All variants detected were classified according to the ACMG/AMP guidelines for interpretation of sequence variants [21], modified by ACGS recommendations [22]. None of the probands had an additional LP/P variant in the genes known to be associated with DCM [1].

Whole genome sequencing (WGS) was used for haplotype analysis and was performed as described previously [23].

Segregation analysis was offered to all relatives of probands with an identified LP/P *TTN* variant. The study included only one relative with the *TTN*:c.12478del, for whom the Sanger sequencing analysis was performed as follows: the region surrounding the variant was amplified using a set of primers (forward primer: 5’ TTGGAGCAAGACAAGCTCACT 3’, reverse primer: 5’ GCACTTTGTGCCTCTTGCTTT 3’), resulting in a PCR product of 428 bp in length. Results were analysed using the latest version of the Geneious^®^ software version 10.2.6.

### Haplotype reconstruction of *TTN*:c.12478del

WGS data were available for six individuals with *TTN*:c.12478del (five probands (P1.1-P5.1) and one first-degree relative with the variant (P4.2.), making these six individuals the patient population. WGS data were also available for 476 individuals (including 36 trios) with non-cardiac referrals identified in the CIGM database, which were used as a control population.

Haplotype analysis was performed only using data from the control population to identify informative single nucleotide polymorphisms (SNPs). Using vcftools [24], only genotype data for SNPs covering a region of approximately 10 Mbp around the locus of the *TTN*:c.12478del variant (chr2:174605481–184605481) with a minor allele frequency above 0.48 were filtered out and used for the further analysis, identifying a total of 801 SNPs at this stage. Using vcftools [24] and a list of obtained SNPs, the same genetic data was collected for the patient population. The genotype data from the control and patient populations were accordingly combined into a single file. In order to identify only tag SNPs, PLINK [25] was used with the command “--indep-pairwise” with a window size of 100, a step size of 10, and an r2 threshold of 0.8, resulting in the identification of 133 tag SNPs. The phase of the alleles on the tag SNPs was defined manually in the Excel spreadsheet [26] using first-degree relatives genotypes. SNPs that were not informative (phase could not be defined) were excluded from further analysis. In addition, manual inspection of the raw genomic data using IGV [27] was performed to also exclude SNPs located in poorly covered or complex regions. The final analysis consisted of 22 tag SNPs used to describe the “disease” haplotype. Haploview [28] was then used to determine the frequency of the “disease” haplotype in the control population to test whether the “disease” haplotype was statistically enriched in the patient population.

Microsatellites and two- and three-nucleotide repeats were used in addition to SNPs to describe the haplotype. Therefore, Ensembl [29] was used to define microsatellites and the USCS Genome Browser [30] was searched for known two- and three-nucleotide repeats mapping to the disease haplotype region. No informative microsatellites were found, but two two-nucleotide repeats (21xTG and 22xAC) and one three-nucleotide repeat (16xATT) were identified mapping to the hg19 reference genome on chromosome 2 at nucleotides 178,070,137–178,070,179, 178,845,703–178,845,747, and 178,736,253–178,736,300 respectively.

### Clinical characterisation

Individual medical histories were collected retrospectively from medical records. When available, data were obtained for baseline 12-lead resting electrocardiogram (ECG), transthoracic echocardiography (TTE), cardiac magnetic resonance imaging (CMR), and biochemical laboratory tests. Categorical variables are expressed as the number of probands with percentages in brackets, while continuous variables are expressed as mean ± standard deviation with the range in brackets.

A three-generation pedigree was constructed for the segregation study.

## Results

### *TTN*tv-s detected in a study cohort

Forty-two unique *TTN*tv-s were identified in 54 probands (Table 1). Most of them (52, 96%) were identified in probands with DCM, one (2%) in a proband with NCC, and one (2%) in a proband with HCM. Three (7%) variants were classified as pathogenic and thirty (71%) as likely pathogenic, all identified in the probands with DCM. The remaining nine variants were classified as variants of uncertain significance and were identified in probands with DCM, NCC, and HCM. Half (21, 50%) of the variants were detected in the A-band, one-third (14, 33.3%) in the I-band, about one-tenth (5, 11.9%) in the M-band, one (2.4%) in the Z-disc, and one (2.4%) in the border exon between the Z-disc and the I-band. Twenty-six (61.9%) were frameshifts, 14 (33.3%) were nonsense, and two were splice site variants, both affecting the symmetric exon. More than half (25, 59.5%) of the variants were novel. The most common variant identified was c.12478del, which affected seven probands referred for DCM. Additional screening of the CIGM database revealed the presence of the variant in a further five non-cardiac referrals. Most other variants were identified in a single proband, with the exception of c.53228_53232del, which was identified in five probands, and c.97260del and c.103030del, which were identified in two probands.

**Table 1:** Rare predicted truncating *TTN* (NM_001267550.2) variants affecting constitutively expressed exons identified in Slovenian probands with cardiomyopathy

Nucleotide change	Protein change	Molecular consequence	Phenotype	No. affected	Region	Classification	Criteria applied	Novel
c.586G > T	p.Glu196*	NonS	DCM	1	Z	VUS	PM2	Y
c.5195dup	p.Thr1733fs	FS	NCC	1	Z/I	VUS	PM2	Y
c.6803_6805delAGTins CAACTGCACCTGAA GGTGCA	p.Glu2268fs	FS	DCM	1	I	LP	PVS1_STR, PM2	Y
c.11398del	p.Leu3800fs	FS	DCM	1	I	LP	PVS1_STR, PM2	Y
c.11808T > A	p.Cys3936*	NonS	DCM	1	I	LP	PVS1_STR, PM2	Y
c.12478del	p.Thr4160fs	FS	DCM	7	I	*P*	PVS1_STR, PS4,PM2	*N*
c.12681del	p.Arg335Trp	FS	DCM	1	I	LP	PVS1_STR, PM2	*N*
c.40,723 + 2T > C	p.?	SS	HCM	1	I	VUS	PVS1_MOD, PM2	Y
c.41725G > T	p.Gly13909*	NonS	DCM	1	I	LP	PVS1_STR, PM2	Y
c.42056del	p.Arg14019fs	FS	DCM	1	I	LP	PVS1_STR, PM2	Y
c.42081del	p.Lys14027fs	FS	DCM	1	I	LP	PVS1_STR, PM2	Y
c.43509del	p.Asp14505fs	FS	DCM	1	I	LP	PVS1_STR, PM2	Y
c.45,322 C > T	p.Arg15108Ter	NonS	DCM	1	I	LP	PVS1_STR, PS4_SUP, PM2	*N*
c.46,236 C > A	p.Cys15412*	NonS	DCM	1	I	LP	PVS1_STR, PM2	*N*
c.46,603 C > T	p.Arg15535Ter	NonS	DCM	1	I	LP	PVS1_STR, PM2	*N*
c.46757dup	p.Met15587fs	FS	DCM	1	I	LP	PVS1_STR, PM2	*N*
c.47639G > A	p.Trp15880*	NonS	DCM	1	A	LP	PVS1_STR, PM2	Y
c.47961del	p.Gly15988fs	FS	DCM	1	A	LP	PVS1_STR, PM2	*N*
c.50,083 C > T	p.Arg16695*	NonS	DCM	1	A	*P*	PVS1_STR, PM2	*N*
c.51,739 + 1G > A	p.?	SS	DCM	1	A	VUS	PVS1_MOD, PM2	Y
c.53228_53232del	p.Val17743fs	FS	DCM	5	A	*P*	PVS1_STR, PS4,PM2	Y
c.58428dup	p.Val19477fs	FS	DCM	1	A	LP	PVS1_STR, PM2	Y
c.62337_62340de**l**	p.Thr20780fs	FS	DCM	1	A	LP	PVS1_STR, PM2	*N*
c.66491_66495dup	p.Tyr22166fs	FS	DCM	1	A	LP	PVS1_STR, PM2	Y
c.68427del	p.Glu22810fs	FS	DCM	1	A	LP	PVS1_STR, PM2	Y
c.68,449 C > T	p.Arg22817*	NonS	DCM	1	A	LP	PVS1_STR, PS4_MOD, PM2	*N*
c.72669del	p.Asp24224fs	FS	DCM	1	A	LP	PVS1_STR, PS4_MOD, PM2	*N*
c.75,469 C > T	p.Arg25157*	NonS	DCM	1	A	LP	PVS1_STR, PM2	*N*
c.81447del	p.Glu27150fs	FS	DCM	1	A	LP	PVS1_STR, PM2	Y
c.82251G > A	p.Trp27417*	NonS	DCM	1	A	LP	PVS1_STR, PM2	Y
c.83748dup	p.Lys27917fs	FS	DCM	1	A	LP	PVS1_STR, PM2	Y
c.86003dup	p.Thr28669fs	FS	DCM	1	A	LP	PVS1_STR, PM2	*N*
c.86093G > A	p.Trp28698*	NonS	DCM	1	A	LP	PVS1_STR, PM2	*N*
c.87341_87343dup	p.Arg29114_Tyr29115insTer	NonS	DCM	1	A	LP	PVS1_STR, PM2	Y
c.90241_90248del	p.Gly30081fs	FS	DCM	1	A	LP	PVS1_STR, PM2	Y
c.96901del	p.Arg32301fs	FS	DCM	1	A	LP	PVS1_STR, PM2	Y
c.97260del	p.Trp32421fs	FS	DCM	2	A	LP	PVS1_STR, PS4_SUP, PM2	Y
c.100919del	p.Gly33640fs	FS	DCM	1	M	VUS	PM2	Y
c.103030del	p.Cys34344fs	FS	DCM	2	M	VUS	PM2,PS4_SUP	Y
c.103360del	p.Glu34454fs	FS	DCM	1	M	VUS	PM2,PM3_STR	*N*
c.104,515 C > T	p.Arg34839*	NonS	DCM	1	M	VUS	PM2	*N*
c.106734dup	p.Ser35579fs	FS	DCM	1	M	VUS	PM2	*N*

A, A-band; DCM, dilated cardiomyopathy; FS, frameshift; I, I-band; HCM, hypertrophic cardiomyopathy; LP, likely pathogenic; M, M-band; N, no; NCC, noncompaction cardiomyopathy; NonS, nonsense; P, pathogenic; SS, splice-site variant; Y, yes; VUS, variant of uncertain significance; Z, Z-disc

### Haplotype reconstruction

Haplotype analysis revealed a region of approximately 2.2 Mbp shared by all six individuals with *TTN*:c.12478del used for analysis. The haplotype was found to be significantly enriched in patients with *TTN*:c.12478del (*p* = 8.217E-45) compared to probands with non-cardiac referrals. The results of the analysis are presented in Table 2.

**Table 2:** Haplotype analysis in five probands with *TTN*:c.12478del (P1.1-P5.1) and one first-degree relative (P4.2)

Marker ID	P1.1	P2.1	P3.1	P4.1	P4.2	P5.1	HAPLOTYPE
rs4894127	G G	G A	A A	G G	G A	G A	/
rs895838	G G	G G	G G	C G	G G	G G	G
rs4893895	T T	T A	T A	T T	T T	T T	T
rs1839152	A A	A G	A G	A A	A A	A G	A
rs2885627	A G	A A	A G	A A	A A	A G	A
rs12623637	A A	G A	A A	A A	A A	A A	A
rs1398967	A G	A G	A G	A G	A A	A A	A
rs10930768	C A	C A	C A	C A	A A	A A	A
rs13422461	C T	C T	C T	C T	T T	T T	T
rs2885984	G A	A A	A A	G A	A A	G A	A
rs62173650	G G	G G	T G	G G	G G	G G	G
21xTG	20|24	20|24	20|24	20|27	20|24	20|27	20
rs6433659	A G	A G	A A	A A	A G	A A	A
rs7423941	A A	A A	A A	A G	A A	A G	A
16xATT	13|16	13|16	13|16	13|14	13|13	13|11	13
22xAC	24|20	24|17	24|20	24|18	24|24	24|19	24
rs2043547	T G	G G	T G	G G	G G	T G	G
rs6726222	T C	T T	T C	T T	T T	T C	T
rs334096	G A	G A	G A	G A	A A	A A	A
rs334122	G A	G A	G A	G A	G G	G G	G
rs334024	G A	G A	G A	A A	A A	A A	A
rs334624	C C	T C	T C	C C	C C	C C	C
variant	T delT	T delT	T delT	T delT	T delT	T delT	T delT
rs4894050	A G	G G	A G	G G	G G	G G	G
rs6433735	C T	C C	C T	C C	C C	C C	C
rs2046775	A C	A A	A A	A C	C C	A A	/

PX.1 (X = family numerical identifier), proband; PX.X, relative of a family X. SNP markers are reported as “allele1” “allele2” identified at the locus. Di- and tri-nucleotide repeat markers are reported as number of repeats per “allele1” “allele2” observed at the locus

### Classification of a *TTN*:c.12478del

The *TTN*:c.12478del, p.(Thr4160fs), is a frameshift variant and is expected to result in a truncation of the titin protein, thereby affecting the function of the protein. Several lines of evidence support the pathogenicity of the variant, as it, first, affects a constitutively expressed exon in the titin I-band, where *TTN*tv-s variants are more frequent in patients with DCM (odds ratio of 19.0) than in the general population, but with a lower risk of DCM than A-band variants (odds ratio 49.8) [8]. Second, the variant is very rare in control populations, having only been identified in one individual from the GnomAD control population [3] and five in the CIGM database. Third, the variant was identified in seven probands with DCM in the CIGM database and in one individual with DCM reported in the ClinVar database (Variation ID: 1403910).

The variant was classified as pathogenic (criteria used: PVS1_STR, PS4, PM2) according to the standards and guidelines for the interpretation of sequence variants established by the ACMG/AMP [21, 31], modified by the ACGS recommendations [22].

### Phenotypic characterization of probands with *TTN*:c.12478del

Detailed medical histories were available for seven probands with *TTN*:c.12478del and DCM, for three out of four of their relatives with the variant, and for one proband with a non-cardiac referral. Summary statistics of the phenotypic findings in the probands are presented in Table 3.

**Table 3:** Clinical characteristics of seven probands with DCM and *TTN*:c.12478del

Category	Probands with TTN:c.12478del
No of probands	7
Male sex	5 (71%)
Age at diagnosis [years]	56.4 ± 10.4 (39–73)
Family history of DCM	2 (29%)
Family history of SCD	0
Baseline clinical evaluation	
NYHA III	5 (71%)
Electrocardiogram	
AF	2 (29%)
PR interval [ms]	188.0 ± 15.9 (164–210)
QRS duration [ms]	97.1 ± 10.5 (80–110)
Abnormal T-waves inversion	6 (86%)
Low QRS voltage limb leads	1 (14%)
Transthoracic echocardiography	
LVEF [%]	25.7 ± 7.1 (14–38)
LVEF <40%	7 (100%)
LVEDV [mL]	201.3 ± 59.5 (134–315)
RV inflow diameter [cm]	3.5 ± 0.5 (2.7–4.3)
TAPSE [cm]	1.9 ± 0.3 (1,4-2.2)
Cardiac magnetic resonance imaging	
LGE - presence of scar	4 (57%)
Biochemistry	
CK [μkat/L]	2.16 ± 0.64 (1.47–3.46)
NT-proBNP [ng/L]	3945 ± 2411 (488–7275)
Events	
NSVT/VT	2 (29%)
aSCD	1 (14%)
ICD	3 (43%)
Heart transplantation	1 (14%)

AF; atrial fibrillation; aSCD, aborted sudden cardiac death; CK; creatine kinase; DCM; dilated cardiomyopathy; EDV, end-diastolic volume; EF, ejection fraction; ICD, implantable cardioverter defibrillator; LGE, late gadolinium enhancement; LV, left ventricle; NYHA, New York Heart Association; NSVT, non-sustained ventricular tachycardia; NT-proBNP, N-terminal prohormone of brain natriuretic peptide; RV, right ventricle; TAPSE, tricuspid annular plane systolic excursion; VT, ventricular tachycardia

On average, the probands were diagnosed with DCM in their sixth decade, with one proband presenting earlier, at the age of 39. Five were male. Two reported a family history of DCM, while none reported a relevant family history of sudden cardiac death. At baseline, two probands had mild symptoms and limitations in normal activities, while five had marked limitations. Arrhythmias were observed in three probands. One had both atrial and ventricular arrhythmias, and two others had either atrial or ventricular arrhythmias. Six probands had abnormal ECG findings. Transthoracic imaging showed an enlarged left ventricle and severely reduced left ventricular ejection fraction (LVEF) in all probands. More than half had subepicardial areas of late gadolinium enhancement (LGE), which were considered likely to be myocardial fibrosis. All probands had significantly elevated levels of NT-proBNP, whereas creatine kinase was within the normal range. One of them experienced sudden cardiac death due to ventricular tachycardia and was resuscitated, received an implantable cardioverter defibrillator (ICD), and later underwent a successful heart transplantation. Two other probands had an ICD implanted, one for primary and one for secondary prevention.

## Discussion

In line with findings in patients with DCM from other populations, the present study shows that (likely) pathogenic *TTN*tv-s represent a significant genetic contributor in the aetiology of DCM in Slovenian patients [1, 2]. In particular, *TTN*tv-s were reported in almost three quarters (73.3%) of the genotype-positive probands with DCM, a proportion three times higher than previously observed [1, 2]. With c.12478del identified in 11.6% of genotype-positive probands with DCM, this study also provides further evidence that rare *TTN*tv-s in the constitutively expressed exons of the proximal I-band region are a relevant cause of DCM.

Extensive research into the exact mechanism of pathogenicity of *TTN*tv-s is ongoing [32]. In addition to earlier reports suggesting insufficiency of untruncated titin [12] and deregulation of translation as a pathomechanism [33], recent studies have provided evidence for a toxic peptide mechanism involving either incorporation of truncated titin into the sarcomere or its accumulation as intracellular aggregates and impaired intracellular protein quality control [12, 34, 35].

The present study’s genetic analysis revealed the presence of rare *TTN*tv-s in the constitutively expressed exons in all titin bands in the patients with cardiomyopathy. In a large case-control study, those *TTN*tv*-*s in the A- and I-band were found to be highly associated with DCM, with an odds ratios of 49.8 for variants in the A-band and 19.0, 19.5, and 32.0 for the I-band, respectively [8]. The disease-causing *TTN*tv-s were detected in the A- and I-band in 44 (73.3%) Slovenian probands with DCM. In the same case-control study, it was found that rare *TTN*tv-s in the constitutively expressed exons located in the Z-disc and M-band were associated with DCM, but the odds ratios were much lower, at 5.3 and 3.7 for the Z-disc and M-band, respectively [8]. In view of this finding and the lack of evidence in the literature, we did not apply the PVS1_STR criterion to the *TTN*tv-s in the M-band and Z-disc and thus classified them as VUS. However, this may change in the near future as studies in large cohorts suggest that rare *TTN*tv-s are enriched across the entire gene in patients with DCM [8, 12–14].

Interestingly, the most common variant identified in the present cohort, *TTN*:c.12478del, does not affect the distal part of the titin but the proximal region of the I-band. It is located in the exon 48, which corresponds to the exon 46 in the N2BA transcript and the exon 45 in the N2B transcript, the predominant cardiac isoforms [16]. This exon is not included in the transcript of the N2A isoform, which is the most abundant isoform in skeletal muscle [7]. Six variants in exon 48 have been reported in the literature in patients with DCM [16, 36–39], indicating the importance of this region in the development of the DCM phenotype. Further, *TTN*:c.12478del is located in the cardiac-specific N2Bus region [10, 11], the importance of which has been demonstrated by mice and zebrafish models [40–44]. Studies in both models have provided valuable insights into why the presence of this region is critical for normal cardiac function. However, in both models the variant was present in a biallelic state, so the findings cannot be directly extrapolated to determine the pathogenicity of DCM, as pathogenic *TTN* variants in patients with DCM are most commonly found in the heterozygous state. Recently, the zebrafish model has been developed to study *TTN*tv-s in the heterozygous state, providing a framework for further functional studies of *TTN*tv-s [45].

Consistent with existing reports of patients with *TTN*tv-s, seven affected individuals with *TTN*:c.12478del presented with DCM, were diagnosed in mid-adulthood [46, 47], and were predominantly male (71%) [15, 36, 46, 48]. All had an LV dysfunction, independent of the presence of fibrosis, which was present in 57% [49]. One proband had a heart transplantation performed and three had an ICD implanted. Arrhythmias were observed in 43% of probands. Previous studies have been inconclusive as to whether the prevalence of atrial and ventricular arrhythmias is increased in patients with DCM and *TTN*tv-s compared to those without *TTN*tv-s [13, 36, 46, 48, 50], ranging up to 43% for atrial [46] and 50% for ventricular [13] arrhythmias. The small sample size may bias our observations of a fairly high incidence. Although only two probands reported a family history of DCM, the haplotype analysis showed that the variant is most likely to be located on the haplotype segment that is common to all individuals with the variant, suggesting that the variant is more likely to have arisen in a common ancestor. Functional studies to determine the effect of the variant on the protein product were beyond the scope of this study.

## Conclusions

Our study elucidated the molecular pathology of *TTN* variants in Slovenian patients with cardiomyopathy and presented the clinical manifestation of the recurrently identified *TTN*:c.12478del, moreover, it contributes to the understanding of the genetic background in this geographical region and highlights the importance of screening for rare *TTN*tv-s in the constitutively expressed exons of the I-band of the gene *TTN* in patients with DCM.

## Data Availability

The datasets used and/or analysed during the current study are available from the corresponding author on reasonable request.
